# Application of Omics Technologies for Cowpea Improvement

**DOI:** 10.1002/pei3.70211

**Published:** 2026-09-15

**Authors:** Junior S. Kamara, Festus O. Olasupo, Abdoul‐Razak Oumarou Mahamane, Katleho S. Selele

**Affiliations:** ^1^ Division of Crop Resources, Ministry of Agriculture Ellen Johnson Sirleaf Ministerial Complex Monrovia Republic of Liberia; ^2^ Plant Breeding Programme Pan African University Life and Earth Sciences Institute (Including Health and Agriculture) Ibadan Nigeria; ^3^ Cocoa Research Institute of Nigeria Ibadan Nigeria; ^4^ Department of Crop Sciences North‐West University (Mahikeng Campus) Mmabatho South Africa

**Keywords:** cowpea, crop improvement, genomics, metabolomics, multi‐omics integration, proteomics, transcriptomics

## Abstract

Cowpea ( *Vigna unguiculata* ) is a vital crop for food security, nutrition, and climate resilience in sub‐Saharan African and other semi‐arid regions. However, its improvement is constrained by the complexity of polygenic traits such as drought tolerance, pest resistance, and seed quality. Conventional breeding, while foundational, remains insufficient to address these challenges at the required pace. Recent advances in multi‐omics technologies, including genomics, transcriptomics, proteomics, and metabolomics, provide new opportunities to dissect complex traits, identify candidate genes, and accelerate the development of resilient, high‐yielding cultivars. This review presents a critical synthesis of current applications of omics technologies in cowpea improvement, highlighting their contributions to stress adaptation, nutritional enhancement, and precision breeding. The review also examines key technical and institutional constraints limiting the adoption of omics‐assisted breeding in cowpea, including inadequate research infrastructure, challenges in multi‐omics data integration, and limited technical capacity across breeding programs in sub‐Saharan Africa. It discusses strategies to address these barriers through regional collaboration, investment in bioinformatics capacity, and the integration of computational approaches into breeding pipelines. Overall, the review concludes that combining multi‐omics technologies with artificial intelligence and machine learning has strong potential to improve genotype–phenotype prediction, accelerate breeding decisions, and support the development of climate‐resilient and nutritionally enhanced cowpea cultivars.

## 1 Introduction

Cowpea ( *Vigna unguiculata* [L.] Walp.) is a drought‐tolerant legume and one of the most important food and forage crops in sub‐Saharan Africa, Southeast Asia, and part of North America and Latin America (Chen et al. 2017). As a climate‐resilient species cultivated on more than 12 million hectares across 50 countries, cowpea contributes significantly to food and nutritional security, smallholder livelihoods, and sustainable farming (Chipeta et al. 2025). Belonging to the family *Fabaceae*, cowpea is a diploid species (2*n* = 2*x* = 22) within the genus *Vigna* and tribe *Phaseoleae* (Diouf 2011). Its adaptability to marginal soils, rapid growth cycle, and tolerance to heat and drought make it a strategic crop to mitigate food shortages in vulnerable regions (Diouf 2011).

Nutritionally, cowpea is a low‐cost source of high‐quality protein (20%–32%), complex carbohydrates, and essential vitamins such as thiamine, riboflavin, niacin, and folates (Shevkani et al. 2025). The grain also provides substantial minerals including iron, zinc, calcium, phosphorus, magnesium, and potassium, alongside amino acids such as lysine and tryptophan that complement cereal‐based diets (Shevkani et al. 2025; Boukar et al. 2015). Cowpea's slow‐digestible starch fraction helps moderate postprandial blood glucose, making it beneficial for diabetic diets (Diouf 2011). Its seeds also contain bioactive compounds including phenolics, flavonoids, and saponins, which have been associated with reduced risks of chronic disease (Diouf 2011). As a gluten‐free, cholesterol‐free, and low‐fat food, cowpea has strong potential in modern health‐conscious diets (Shevkani et al. 2025). Beyond its nutritional value, cowpea plays a central role in sustainable farming systems through biological nitrogen fixation, improved soil fertility, and compatibility with cereal intercropping systems (Shevkani et al. 2025). In addition, cowpea haulms serve as high‐quality livestock feed, strengthening crop–livestock integration (Shevkani et al. 2025; Boukar et al. 2015).

Despite its importance, cowpea production faces substantial biotic and abiotic constraints. Major insect pests such as aphids, thrips, pod borers, and bruchids, along with fungal diseases (Fusarium wilt, powdery mildew) and viral infections (cowpea mosaic virus, cowpea aphid‐borne mosaic virus) cause significant yield losses (Ribeiro et al. 2023). Abiotic stresses, including recurrent drought, heat, salinity, and low soil fertility, also reduce productivity in smallholder farming systems where external inputs are limited (Nyaga and Njeru 2020). Post‐harvest storage losses, particularly from bruchid infestations, remain a persistent challenge for food and nutritional security.

Conventional breeding programs have focused on improving early maturity, consumer‐preferred seed types, and resistance to pests and diseases (Alemu et al. 2016; Chen et al. 2017). However, genetic improvement has progressed more slowly than needed because many priority traits, including yield stability, abiotic stress tolerance, and nutritional quality, are quantitatively inherited, controlled by multiple loci, and strongly influenced by genotype × environment interactions. As a result, conventional breeding, which relies heavily on phenotypic selection, often requires lengthy breeding cycles and provides limited precision for identifying superior genotypes carrying favorable allelic combinations. Although genomic resources for cowpea have expanded substantially in recent years, their effective integration into routine breeding programs remains limited, particularly in resource‐constrained settings (Boukar et al. 2015). Furthermore, while marker‐assisted selection has been successful for traits controlled by major‐effect loci, its application is less effective for complex polygenic traits, highlighting the need for more comprehensive genomics‐enabled approaches capable of capturing the genetic architecture of quantitative traits (Chen et al. 2017).

**FIGURE 1:**
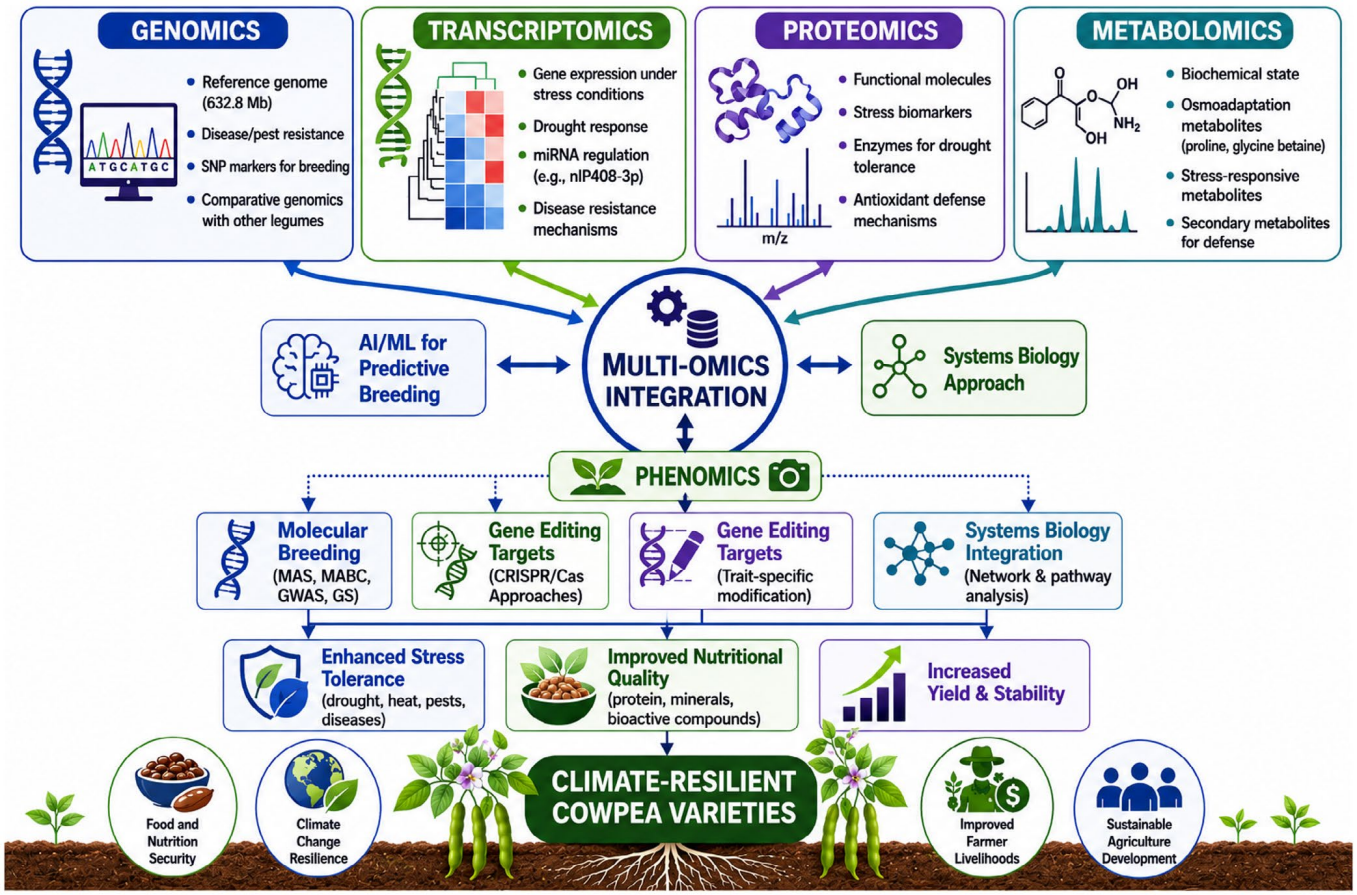
Conceptual framework for integrating multi‐omics technologies in cowpea improvement. The framework illustrates how genomics, transcriptomics, proteomics, metabolomics, phenomics, systems biology, and AI/ML can be integrated to support molecular breeding, genome editing, genomic selection, and predictive breeding for climate‐resilient, nutritionally improved, and stress‐tolerant cowpea cultivars. The framework was developed by the authors based on the literature reviewed in this article and prepared using BioRender.com.

Recent advances in multi‐omics technologies, including genomics, transcriptomics, proteomics, and metabolomics, have provided transformative tools to overcome many of the limitations of conventional cowpea breeding. High‐quality genome assemblies, combined with transcriptomic datasets, have enabled the identification of candidate genes associated with drought tolerance, nutrient accumulation, and disease resistance (Zhang, Zhang, et al. 2022). Proteomic and metabolomic analyses have further explained regulatory networks, stress‐responsive pathways, and bioactive metabolites underlying nutritional quality and environmental adaptation (Makhumbila et al. 2022). When integrated through systems biology frameworks, these complementary datasets offer a comprehensive understanding of the molecular architecture of complex traits, linking genomic variation with gene expression, protein function, and metabolic regulation. Such integration enhances the identification of favorable alleles, elucidates regulatory networks, and provides a robust foundation for precision breeding and predictive improvement of cowpea. The conceptual framework for this integrated approach is presented in Figure 1.

Although omics technologies have substantially advanced the understanding of cowpea biology, most applications remain confined to single‐platform studies. Integrated analyses combining genomics, transcriptomics, proteomics, and metabolomics have revealed stress‐responsive regulatory networks, genetic diversity, and symbiotic nitrogen fixation, yet their potential for breeding translation is still underutilized (Zhang, Zhang, et al. 2022). Key gaps persist: multi‐omics integration for dissecting complex agronomic and nutritional traits is still limited, metabolomic characterization of quality‐related traits remains underexplored, and the translation of omics‐derived discoveries into routine breeding pipelines has been relatively slow. Addressing these limitations will require stronger integration of existing genomic resources with high‐throughput phenotyping, advanced bioinformatics and artificial intelligence‐based analytical frameworks, and rigorous field validation to convert multi‐omics discoveries into practical breeding applications. Therefore, this review provides an overview of recent applications of omics technologies in cowpea research, exploring how these tools can accelerate the dissection of complex traits and facilitate the development of climate‐resilient, nutrient‐dense cowpea varieties. It aims to demonstrate how integrated multi‐omics approaches can unlock the crop's genetic potential and reinforce its contribution to sustainable agriculture in the face of climate change and growing population pressures.

## 2 Overview of Omic Technologies

**FIGURE 2:**
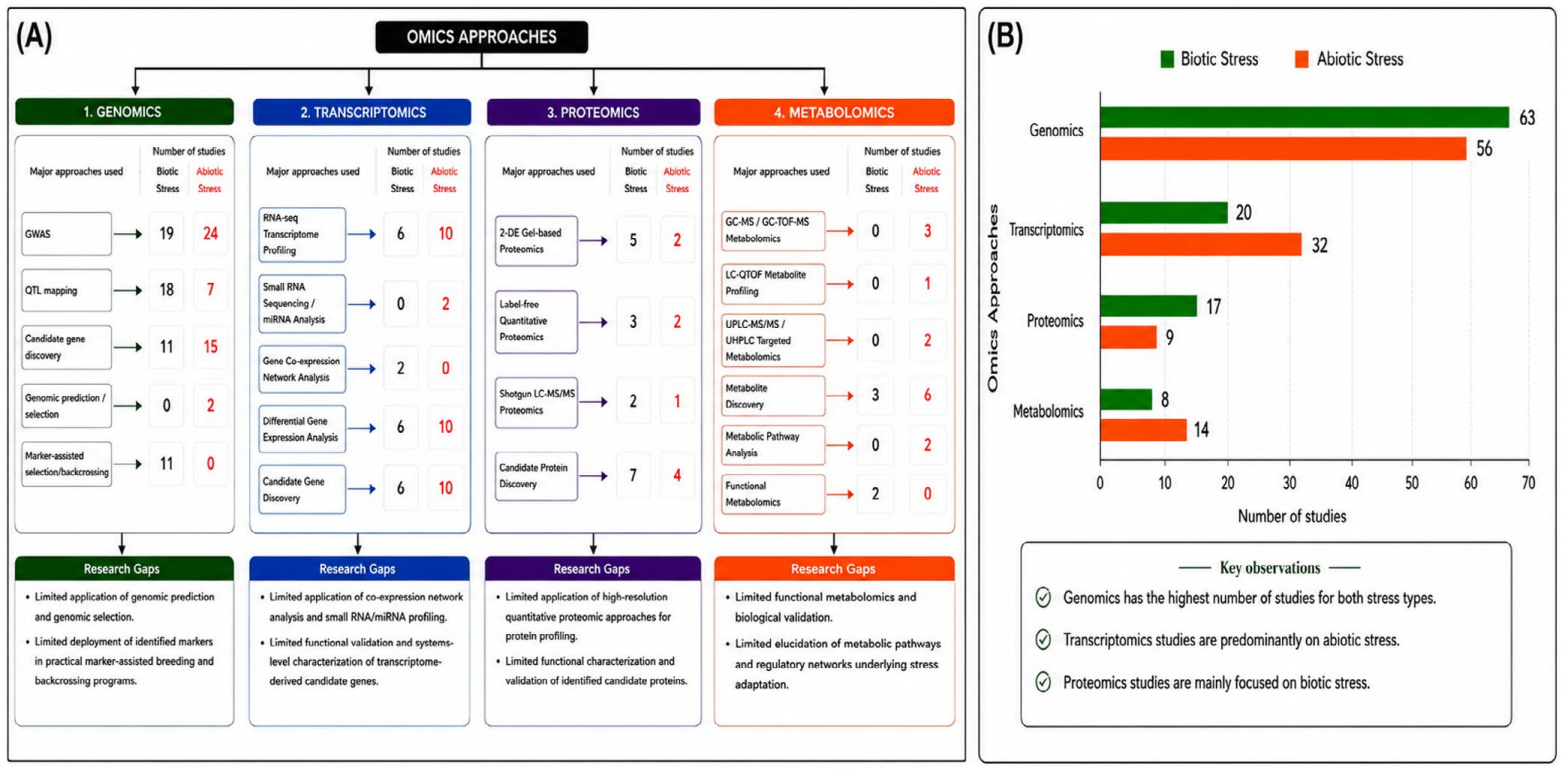
Comparative overview of omics approaches applied to cowpea improvement under biotic and abiotic stresses. (A) Major genomics, transcriptomics, proteomics, and metabolomics approaches, together with the corresponding number of studies and identified research gaps. (B) Comparative distribution of studies investigating biotic and abiotic stresses across the four omics approaches. The figure was created using BioRender based on a systematic literature survey of Web of Science and Scopus databases covering studies published between 2010 and 2025 using key words including (“cowpea drought GWAS”), (“cowpea disease resistance GWAS”), (“cowpea SNP diversity”), (“cowpea population structure”), (“cowpea genomic selection”), (“cowpea genomic prediction”), (“cowpea transcriptome drought”), (“cowpea RNA‐seq drought”), (“cowpea dehydration transcriptome”), (“cowpea heat stress RNA‐seq”), (“cowpea salinity transcriptome”), (“cowpea aphid RNA‐seq”), (“cowpea disease transcriptomics”), (“cowpea metabolomics drought”), (“cowpea metabolome stress”), (“cowpea salinity metabolomics”), (“cowpea heat metabolomics”), (“cowpea metabolomics insect resistance”), (“cowpea thrips metabolomics”), (“cowpea bruchid metabolomics”), (“cowpea proteomics disease resistance”), (“cowpea drought proteomics”), (“cowpea salt stress proteomics”), and (“cowpea heat stress proteomics”). Numbers represent the frequency of studies identified under the defined search strategy and serve as indicators of the relative research emphasis across omics approaches in cowpea.

Omics technologies comprise a set of high‐throughput approaches that characterize biological systems at successive molecular levels: genomics (DNA sequence and variation), transcriptomics (RNA expression and regulation), proteomics (protein abundance, modifications, and interactions), and metabolomics (small‐molecule profiles). Together, these layers generate complementary datasets that reveal how genetic variation shapes molecular function and adaptive responses to environmental stress, providing a systems‐level foundation for crop improvement (Yang et al. 2021). A comparative synthesis of published studies highlights the relative emphasis and methodological diversity across omics approaches in cowpea research, revealing distinct patterns of application under biotic and abiotic stress conditions (Figure 2).

## 3 Genomics

Genomics is the study of the structure, function, evolution, and mapping of genomes, providing the foundation for linking genetic variation to traits of interest in crop improvement (Zhang 2023). This approach enables breeders to dissect complex traits, accelerate selection, and introduce beneficial alleles with greater precision compared to conventional breeding. Key genomic approaches include Quantitative Trait Loci (QTL) Mapping, Genome‐Wide Association Studies (GWAS), Marker‐Assisted Selection (MAS), Genomic Selection (GS), and Genome Editing (Kumar et al. 2024). QTL mapping enables the identification of genomic regions that control complex traits such as yield, stress tolerance, and disease resistance, although its resolution is sometimes limited (Cooper et al. 2009). Genome‐Wide Association Studies (GWAS) build on natural variation within diverse populations to detect marker‐trait associations at higher resolution, making them particularly useful for dissecting polygenic traits (Susmitha et al. 2023). Marker‐Assisted Selection (MAS) applies markers linked to major genes or QTLs to accelerate breeding decisions, especially for traits with large effects and simple inheritance patterns (Susmitha et al. 2023). Genomic Selection (GS), in contrast, employs genome‐wide markers to predict breeding values without requiring prior knowledge of the specific loci underlying a trait, thus offering greater efficiency for improving complex traits (Alemu et al. 2024). More recently, genome editing tools such as CRISPR/Cas have enabled precise modifications of target genes, creating novel allelic variants that enhance yield, nutritional quality, or stress resilience (Abdallah et al. 2015).

## 4 Transcriptomics

Transcriptomics refers to the comprehensive study of all RNA molecules transcribed from a genome in a given cell, tissue, or developmental stage, including both messenger RNAs and non‐coding RNAs (Tyagi et al. 2022). Unlike genomics, which provides a static view of an organism's DNA, transcriptomics captures the dynamic patterns of gene expression that vary across time, developmental stages, tissues, and environmental conditions (Tyagi et al. 2022). This makes it a powerful approach for identifying genes and pathways involved in growth, development, stress responses, and the production of secondary metabolites (Figure 2). Advances in sequencing technology have transformed transcriptomic research. Early methods such as microarrays provided insights into known genes but were limited in sensitivity and scope (Dai and Shen 2022). The emergence of high‐throughput RNA sequencing (RNA‐seq) has made it possible to profile entire transcriptomes with high sensitivity and accuracy, even in the absence of a reference genome (Dai and Shen 2022). RNA‐seq allows researchers to quantify gene expression, detect alternative splicing events, discover novel transcripts, and identify sequence polymorphisms. In crop improvement, transcriptomic approaches have been widely applied to characterize gene expression under abiotic and biotic stresses, identify candidate genes for tolerance and resistance, and uncover regulatory networks that control important agronomic traits (Varadharajan et al. 2025). Studies using RNA‐seq have revealed key regulators of stress responses, biosynthetic pathways for bioactive compounds, and developmental processes that can be targeted in breeding programs (Hossain et al. 2018; Liu et al. 2024).

## 5 Proteomics

Proteomics is the large‐scale study of proteins, focusing on identifying, quantifying, and characterizing the protein complement of cells, tissues, and organs under defined conditions (Aslam et al. 2017). Since proteins are the primary effectors of cellular processes, proteomics provides a functional link between the genome and phenotype, offering insights into how plants regulate growth, development, and stress responses (Kosová et al. 2011). Unlike genomics, this reveals genetic potential, proteomics captures the dynamic changes in protein abundance, localization, interactions, and post‐translational modifications that directly determine plant performance (Mustafa and Komatsu 2021).

In crop improvement, proteomics has been widely applied to study responses to abiotic stresses such as drought, salinity, flooding, and heavy metals, as well as biotic stresses including pathogens and pests (Varadharajan et al. 2025). Comparative proteomic analyses allow the identification of stress‐responsive proteins and pathways, helping to unravel the molecular mechanisms underlying tolerance (Kosová et al. 2011). Advances in mass spectrometry and gel‐free proteomic platforms have enhanced protein detection sensitivity, enabling more comprehensive profiling of complex plant proteomes (Vadivel 2015). Subcellular proteomics further refines this approach by mapping proteins within organelles, while studies on post‐translational modifications such as phosphorylation, glycosylation, and ubiquitination reveal additional layers of regulatory control during stress adaptation (Mustafa and Komatsu 2021).

By identifying key stress‐responsive proteins and linking them to functional genes, proteomics facilitates the discovery of candidate markers for marker‐assisted selection and informs genome editing strategies (Varadharajan et al. 2025). Although challenges remain, including protein extraction complexity, data interpretation, and the low abundance of certain regulatory proteins, proteomics continues to emerge as an indispensable tool complementing genomics and transcriptomics in modern breeding pipelines.

## 6 Metabolomics

Metabolomics, also known as metabolite profiling, is the comprehensive study of small molecules (metabolites) in biological systems, providing insights into cellular processes, metabolic networks, and organismal responses to genetic and environmental changes (Salam et al. 2023). Metabolites including sugars, amino acids, lipids, nucleotides, secondary metabolites, and hormones play critical roles in energy production, growth, development, stress adaptation, and interactions with the environment (Salam et al. 2023).

Metabolomics has emerged as a key tool in plant stress biology, helping elucidate mechanisms underlying drought, salinity, and heat tolerance (Carrera et al. 2021). In crop improvement, metabolomics assists breeders by linking metabolites to phenotypic traits and quantitative trait loci (QTLs). Metabolite‐based markers (mQTLs) allow selection for desirable traits such as stress tolerance, yield, fruit quality, and nutritional value (Rouphael et al. 2016; Templer et al. 2017). By integrating metabolomics into breeding pipelines, it is possible to accelerate the development of high‐yielding, resilient, and nutritionally enhanced crops, while reducing dependency on inputs like pesticides, fertilizers, and water (Christ et al. 2018).

## 7 Advances in Cowpea Genomics

Genomics provides the foundational layer of multi‐omics research in cowpea, offering a structural blueprint of the genome and enabling the dissection of complex traits through high‐resolution molecular tools (Lin and Lane 2017). As a drought‐resilient legume cultivated across diverse agroecological zones, cowpea has attracted significant genomic research aimed at improving yield, stress tolerance, and nutritional quality (Boukar et al. 2016). Early genomic studies underscored the importance of genome characterization for breeding applications (Boukar et al. 2015). The major genomic resources developed for cowpea and their specific breeding applications are summarized in Table 1.

**TABLE 1:** Summary of major cowpea genomic resources, their key features, and applications in molecular breeding.

Resource (type)	Key features	Primary applications	References
Reference genome: IT97K‐499‐35	High‐quality assembly (~519 Mb; > 95% coverage); primary coordinate system for cowpea genetics	Gene discovery, synteny, GWAS read mapping	Lonardi et al. 2019
Cowpea pan‐genome	Multi‐accession assemblies; ~35,436 pan genes (26,026 core, 4963 variable)	Adaptation/stress gene discovery; parent selection	Liang et al. 2024
SNP panels (iSelect/core)	Medium‐to‐high density (~50 K SNPs); validated across global germplasm	Diversity analysis, GWAS, parent selection, marker‐assisted breeding	Muchero et al. 2009; Muñoz‐Amatriaín et al. 2017
QTL mapping (bi‐parental populations, diversity panels)	Loci for flowering, seed traits, drought tolerance, pest/disease resistance	Marker‐trait associations, diagnostic markers, candidate gene nomination.	Andargie et al. 2014; Ravelombola et al. 2021; Sodo et al. 2025
Marker‐assisted backcrossing (MABC)	Validated markers flanking major QTLs (Striga, nematode, aphid resistance)	Rapid introgression of resistance alleles; pyramiding of loci	Batieno et al. 2016
Genomic selection (GS)	Epistasis‐aware predictive models; improved prediction of complex traits	Accelerated genetic gain, optimized training populations	Olatoye et al. 2019

A major milestone in cowpea genomics was the development of a high‐quality reference genome for the African cultivar IT97K‐499‐35, assembled using PacBio long‐read sequencing, Bionano optical mapping, and high‐density genetic linkage maps (Lonardi et al. 2019). This assembly spans approximately 519 Mb. Publicly accessible through platforms such as phytozome and Ensembl Plants, this resource has catalyzed molecular breeding, comparative genomics, and gene discovery efforts. More recently, Liang et al. (2024) constructed the first cowpea pan‐genome using de novo assemblies from multiple cultivated accessions representing diverse subpopulations. The pan‐genome comprises 35,436 genes, including 26,026 core and 4963 variable genes. Functional enrichment analysis revealed that the variable gene set is significantly associated with pathogen defense, abiotic stress response, and secondary metabolism.

Quantitative trait loci (QTL) mapping has also been instrumental in identifying genomic regions linked to agronomic traits. Muchero et al. (2009) mapped 12 QTLs associated with seedling‐stage drought tolerance and maturity using a recombinant inbred line (RIL) population derived from IT93K503‐1 × CB46. Ten drought‐response QTLs were consistently detected across greenhouse and field trials, located on linkage Groups 1, 2, 3, 5, 6, 7, 9, and 10, explaining 4.7% to 24.2% of the phenotypic variance. Notably, QTLs Dro‐3 and Dro‐4 showed high stability and significance across environments, while Dro‐8 emerged as a stable locus for drought‐induced senescence. Two maturity‐related QTLs, Mat‐1 and Mat‐2, mapped on linkage Groups 7 and 8, respectively, accounted for up to 28.9% of the variance and were reproducible across multiple experiments. Similarly, Sodo et al. (2025) mapped QTLs for grain yield and associated traits using a RIL population derived from RP270 × CB27. Their analysis, employing a single‐seed descent method under normal rainfed conditions, identified genomic regions linked to seed size, pod number, days to flowering, and peduncle development. Seven major QTLs were identified, each explaining over 10% of the phenotypic variance. These loci offer valuable targets for improving harvest index and yield stability in low‐input environments particularly in sub‐Saharan African.

Complementing QTL studies, genome‐wide association studies (GWAS) have refined trait mapping by utilizing natural variation across diverse germplasm. Wu et al. (2021) applied high‐throughput physiological phenotyping and GWAS to investigate stomatal behavior under progressive soil drought. Two key traits (critical soil moisture threshold for stomatal closure (θcri) and rate of stomatal closure (KTr)) were evaluated across 106 accessions. GWAS identified 13 significant SNPs for θcri and 64 for KTr, distributed across multiple chromosomes. Candidate genes included E3 ubiquitin ligases, late embryogenesis abundant (LEA) proteins, NAC and MYB transcription factors, zinc finger proteins, all implicated in drought response pathways. These findings underscore the polygenic nature of stomatal regulation and offer molecular targets for improving water‐use efficiency in cowpea. A detailed summary of these and other recent trait‐linked genomic discoveries, including findings related to yield, stress tolerance, and seed quality, is presented in Table 2. Similarly, Wu et al. (2024) conducted a large‐scale GWAS and diversity analysis across 373 cowpea accessions, identifying 76,114 polymorphic SNPs and identified a total of 56 significant quantitative trait nucleotides (QTNs) for nine traits, with pod length (25 QTNs), antioxidant content, dry pod color, flower color, and maturity among the most represented. Candidate genes linked to these traits included glycosyl hydrolases, leucine‐rich repeat proteins, AT‐hook DNA‐binding proteins, and photosystem II components.

**TABLE 2:** Summary of trait‐linked discoveries in cowpea related to yield, biotic stress, and abiotic stress, based on GWAS and SNP diversity studies.

Study/authors	Year	Approach	Key findings	Relevance
Liang et al.; Lonardi et al.	2023	Pan‐genome (7 assemblies)	~26,000 core genes; ~4900 non‐core genes (stress‐related); > 5 M SNPs; large SVs	Foundation for gene discovery, diversity mapping
Chen et al.	2023	GWAS for seed protein content	Significant loci linked to protein content	Nutritional quality improvement
Edema et al.	2023	Parental line SNP diversity (Uganda program)	Genetic diversity among breeding parents	Crossing strategy optimization
Wu et al.	2024	High‐quality assemblies + resequencing (344 accessions)	39 loci for 10 agronomic traits; 541 domestication/improvement loci; pod‐shattering linked to stress loci	Guides breeding for yield, pod traits, stress adaptation
Wu et al.	2024	GWAS in global diversity panel (373 accessions)	56 QTNs for 9 traits (pod length, seed weight, antioxidant content, etc.); hotspots identified	Multi‐trait improvement potential
Potts et al.	2024	USDA germplasm SNP analysis	Genetic structure and diversity characterized	Informs parental line use in breeding
Ongom et al.	2024	Breeding progress analysis	Demonstrated genetic gains in yield using genomics tools	Shows practical impact of genomic integration
Ravelombola et al.	2025	GWAS for drought tolerance (331 genotypes)	Major SNP cluster on Chr 5 (~210 kb, 196 SNPs); loci on Chr 1, 3, 5, 11	Molecular markers for drought‐resilient lines

Together, cowpea genomics has progressed from a single reference genome to pan‐genomic and association mapping studies that link genomic variation to yield, biotic resistance, and abiotic stress tolerance. These resources are transforming breeding strategies by providing SNP markers, candidate gene sets, and predictive models, laying the foundation for integration with transcriptomics, proteomics, and metabolomics in a systems‐level approach to cowpea improvement.

## 8 Advances in Cowpea Transcriptomics

Transcriptomics has become an important approach for understanding the molecular mechanisms underlying stress adaptation and resistance in cowpea. The development of high‐throughput RNA sequencing technologies has enabled the identification of genes, signaling pathways, transcription factors, and regulatory networks associated with both biotic and abiotic stresses (Ferreira‐Neto et al. 2021). Recent transcriptomic studies have generated important insights into stress tolerance, development, and quality improvement in cowpea (Table 3).

**TABLE 3:** Major transcriptomic studies in cowpea and their implications for stress tolerance, development, and quality improvement.

Stress/trait investigated	Transcriptomic approach	Major findings	Breeding/functional significance	References
Pod length development	Transcriptomics, GWAS, and QTL mapping	Hormone and sugar signaling pathways	Yield improvement resources	Xu et al. 2016
Chilling stress	miRNA–mRNA transcriptomics	Chilling‐responsive regulatory networks	Cold tolerance regulation	Zuo et al. 2018
Anthocyanin accumulation	RNA‐seq	Flavonoid biosynthesis and MYB regulation	Nutritional quality improvement	Li et al. 2020
Root dehydration stress	Integrated transcriptomics	Raffinose and inositol osmoprotection pathways	Targets for osmotic stress tolerance	Ferreira‐Neto et al. 2021
Root dehydration stress	RNA‐seq	ABA signaling, ROS detoxification, NAC/WRKY/DREB activation	Candidate genes for drought tolerance	Ferreira‐Neto et al. 2022
Drought/dehydration stress	WRKY transcriptome profiling	WRKY‐mediated drought signaling	Understanding drought transcriptional regulation	Matos et al. 2022
Drought and heat stress	Functional transcriptomics	VuDREB2A‐mediated stress adaptation	Abiotic stress breeding target	Kumar et al. 2022
Drought stress	Small RNA and transcriptome analysis	ABA signaling and drought‐responsive miRNAs	miRNA‐mediated drought tolerance	Mishra et al. 2021
Root‐knot nematode resistance	Transcriptomics and proteomics	Defense pathway activation	Nematode resistance breeding	Ribeiro et al. 2022
Salt stress	RNA‐seq	Ion transport and antioxidant pathways	Candidate genes for salinity tolerance	Kang et al. 2023
Multiple abiotic stresses	NAC transcriptome characterization	Stress‐responsive VuNAC genes	Broad‐spectrum stress adaptation	Srivastava et al. 2022
Viral defense responses	Comparative transcriptomics	Conserved antiviral regulatory networks	Virus resistance mechanisms	Borges‐Martins et al. 2023
Resistance to *Megalurothrips usitatus*	Transcriptomics and metabolomics	Jasmonic acid and flavonoid pathways	Pest resistance improvement	Li et al. 2023
High relative humidity stress	RNA‐seq	Hormone and jasmonic acid signaling	Environmental adaptation pathways	Krylova et al. 2024
Viral infection and root dehydration	RNA‐seq and TLP analysis	Thaumatin‐like stress‐responsive proteins	Multifunctional stress‐resistance genes	Jesús‐Pires et al. 2024
Fusarium wilt resistance	WRKY transcriptomics	WRKY‐mediated immune responses	Disease resistance improvement	Hao et al. 2024
Fusarium wilt resistance	RNA‐seq and WGCNA	Melatonin‐induced defense signaling	Novel resistance pathways	Gan et al. 2024
Postharvest senescence	Transcriptomics and metabolomics	Chlorophyll degradation and hormone signaling	Shelf‐life improvement	Liu et al. 2024
CABMV resistance	RNA‐seq	Phenylpropanoid and flavonoid activation	Antiviral defense mechanisms	de Andrade Luz et al. 2026
Anthocyanin biosynthesis	Transcriptomics and metabolomics	MYB–MBW regulatory complexes	Pigmentation enhancement	Yang et al. 2025
Leafminer resistance	Transcriptomics and metabolomics	Defense‐associated transcriptional networks	Pest resistance breeding	Wang et al. 2026

Under biotic stress conditions, transcriptomic studies have shown that cowpea activates rapid and coordinated defense responses involving hormone signaling, ROS detoxification, receptor‐like kinases, resistance genes, and transcription factors. Comparative RNA‐seq analysis of resistant cowpea genotypes infected with Cowpea aphid‐borne mosaic virus (CABMV) and Cowpea severe mosaic virus (CPSMV) revealed strong activation of jasmonic acid and ethylene biosynthesis pathways, MAPK signaling, aquaporins, receptor‐like kinases, and NBS‐LRR resistance genes during the early stages of infection (Borges‐Martins et al. 2023). The study further showed that AP2‐ERF, WRKY, and MYB transcription factors were central regulators of the antiviral response, highlighting the importance of early transcriptional reprogramming in cowpea viral resistance. Similarly, Ribeiro et al. (2022) investigated cowpea resistance to the root‐knot nematode Meloidogyne incognita and demonstrated that resistance is associated with the coordinated activation of NBS‐LRR resistance genes, WRKY transcription factors, jasmonic acid‐mediated defense signaling, protease inhibitors, ROS detoxification pathways, and genes involved in cell wall reinforcement and cytoskeleton organization. These mechanisms likely restrict nematode feeding and development within infected roots.

Transcriptomics has also improved understanding of insect resistance in cowpea. Comparative RNA‐seq analysis of resistant and susceptible near‐isogenic lines infested with cowpea aphid ( *Aphis craccivora* ) revealed that resistant plants possess both constitutive and inducible defense mechanisms (MacWilliams et al. 2023). Resistance was associated with candidate genes such as RGA4 homologs, leucine‐rich repeat receptor‐like kinases, isoflavone reductases, phosphatases, and genes involved in salicylic acid signaling and resource allocation. Similarly, the study of cowpea resistance to the American serpentine leafminer ( *Liriomyza trifolii* ) in south China revealed rapid activation of jasmonic acid signaling, MYC2 transcription factors, MAPK pathways, flavonoid biosynthesis, lignin biosynthesis, and oxidative stress responses in resistant genotypes (Wang et al. 2026). These findings demonstrated that cowpea insect resistance depends on coordinated hormonal regulation and secondary metabolite biosynthesis pathways.

Abiotic stress transcriptomics in cowpea has focused mainly on drought, salinity, heat, and chilling stress. Transcriptome profiling of drought‐tolerant cowpea roots during dehydration stress revealed that extensive transcriptional reprogramming occurs before visible physiological damage appears (Ferreira‐Neto et al. 2022). Early drought responses were dominated by signaling pathways, WRKY and ERF transcription factors, MAP kinases, receptor‐like kinases, ROS detoxification genes, and defense‐related pathways, whereas prolonged stress induced aquaporins, glutathione S‐transferases, cytochrome P450s, NAC transcription factors, and specialized metabolic pathways associated with long‐term stress adjustment. Furthermore, Matos et al. (2022) demonstrated the important role of WRKY transcription in cowpea drought adaptation by identifying several key genes (VuWRKY18, VuWRKY21, and VuWRKY75) that were strongly induced under root dehydration stress, with drought‐tolerant genotypes showing earlier and stronger expression than sensitive genotypes. These findings suggested that rapid activation of WRKY‐mediated transcriptional networks contributes significantly to drought tolerance in cowpea. The importance of inositol and raffinose family oligosaccharide pathways in dehydration tolerance was further demonstrated by the strong induction of genes involved in phosphoinositide signaling, raffinose biosynthesis, membrane remodeling, osmoprotection, and ROS scavenging under drought stress conditions (Ferreira‐Neto et al. 2021).

Transcriptomic analyses have also provided important insights into salinity and heat stress tolerance in cowpea. Comparative RNA‐seq analysis of salt‐tolerant and salt‐sensitive germplasms identified thousands of salt‐responsive genes involved in ion transport, osmotic adjustment, oxidative stress response, phenylpropanoid biosynthesis, and secondary metabolism. Candidate genes such as potassium transporter 6 and EXO70 were proposed as important regulators of salt tolerance (Kang et al. 2023). In transgenic cowpea overexpressing VuDREB2A‐CA, transcriptomic analysis revealed strong induction of stress‐responsive transcription factors, antioxidant enzymes, transporters, and osmoprotective genes, confirming the central role of VuDREB2A in regulating drought and heat stress responses (Kumar et al. 2022).

Beyond drought and salinity, transcriptomics has also improved understanding of chilling injury in cowpea. Integrated transcriptome and small RNA sequencing analyses of vegetable cowpea pods exposed to chilling stress identified genes and miRNAs associated with membrane stability, lipid metabolism, oxidative stress, ethylene signaling, heat shock proteins, and post‐transcriptional regulation (Zuo et al. 2018). The study demonstrated that chilling tolerance is regulated through highly coordinated transcriptional and miRNA‐mediated regulatory networks.

## 9 Advances in Cowpea Proteomics

Although still developing compared with genomics and transcriptomics, cowpea proteomics provides unique insights into protein‐level mechanisms of resistance, tolerance, and adaptation to biotic and abiotic stresses, enabling identification of stress‐responsive proteins, biomarkers, and regulatory pathways (Ribeiro et al. 2023).

Proteomic studies have revealed the dynamic protein changes during interactions with pathogens and pests. In root‐knot nematodes (*Meloidogyne incognita*) infestations, resistant cultivars rapidly accumulated antioxidant enzymes and pathogenesis‐related (PR) proteins, while susceptible lines showed weaker responses (Oliveira et al. 2012). Subsequent work identified defense proteins such as chitinases, β‐1,3‐glucanases, peroxidases, protease inhibitors, and enzymes of ethylene and flavonoid pathways, which collectively restricted nematode feeding site establishment through ROS regulation, cell wall reinforcement, and programmed cell death (Oliveira et al. 2014; Villeth et al. 2015). More recent large‐scale proteomic profiling catalogued over 2300 proteins under nematode stress, linking defense to cytoskeletal proteins, lipid signaling enzymes, and phytoalexin biosynthesis (Ribeiro et al. 2022). When nematode and drought stress occurred simultaneously, resistant plants induced thaumatin‐like proteins and hormone cross‐talk between jasmonic acid and abscisic acid (Ribeiro et al. 2023). Collectively, these studies show that nematode resistance relies on coordinated antioxidant defense, PR protein induction, hormone signaling, and metabolic adjustments.

Proteomics has also clarified cowpea responses to fungal and viral diseases. In anthracnose (*Colletotrichum gloeosporioides*), resistant genotypes upregulated PR proteins, cysteine proteases, heat shock proteins, and enzymes of the jasmonic acid pathway, alongside antioxidant proteins such as SOD and thioredoxin (Moura et al. 2014). Studies of cowpea severe mosaic virus (CPSMV) revealed that early infection suppressed host defense proteins, while resistant genotypes restored proteome balance through selective activation of photosynthesis, chaperones, and proteasome pathways (Paiva et al. 2016; Varela et al. 2017). Abiotic stress such as salinity before infection compromised resistance by disturbing photosynthesis and redox regulation (Varela et al. 2019). Integrated proteomics–small RNA approaches further demonstrated that antiviral resistance involved ROS‐regulating enzymes, protease inhibitors blocking viral polyprotein processing, and miRNA‐mediated regulation of cytoskeletal proteins and RNA helicases (Souza et al. 2020; Martins et al. 2020). Across fungal and viral studies, proteomics consistently emphasizes the central role of oxidative burst, redox regulation, and proteome balance in restricting pathogen growth and supporting resistance.

Proteomics has also been used to dissect cowpea adaptation to salinity, drought, and heat. Under salt stress, tolerant cultivars maintained energy metabolism and photosynthesis by upregulating proteins such as rubisco activase, oxygen‐evolving enhancer proteins, ATP synthase, and ion transporters, whereas sensitive cultivars showed strong downregulation of these proteins (De Abreu et al. 2014; Mini et al. 2019).

Drought proteomics revealed contrasting responses between tolerant and sensitive genotypes. Tolerant cultivars upregulated glutamine synthetase, chaperonins, malate dehydrogenase, HSPs, and proteasome subunits to maintain photosynthesis and protect proteins from damage, while sensitive cultivars showed a decline of photosynthetic proteins (Lima et al. 2019). Ribeiro et al. (2023) further showed that drought also triggered antioxidant enzymes, ABA and JA signaling proteins, and secondary metabolism pathways such as flavonoid biosynthesis, underscoring the multifaceted defense network cowpea employs under water deficit.

Heat stress studies revealed protective proteins in both seeds and leaves. Imbibed seeds accumulated LEA proteins, small heat shock proteins, and superoxide dismutases that stabilize macromolecules and regulate ROS during germination (Zhang and Li 2015). In leaves, tolerant genotypes expressed broad sets of HSPs, molecular chaperones, ROS scavengers, and protein repair enzymes, while downregulating photosynthesis‐related proteins to prioritize survival. This adaptive proteomic reprogramming supported faster recovery of photosystem II and better yield stability under heat (Selinga et al. 2022).

Collectively, proteomic studies of salinity, drought, and heat stress reveal that tolerance depends on maintaining energy metabolism, photosynthesis, antioxidant protection, and protein stability, underscoring the multifaceted defense networks cowpea employs under adverse environments.

## 10 Advances in Cowpea Metabolomics

Metabolomics enables profiling of primary and secondary metabolites under diverse conditions, identifying compounds that explain physiological responses and serve as markers for breeding resilient, high‐quality cowpea varieties (Zhang, Zhang, et al. 2022). One of the most explored areas is stress adaptation. Goufo et al. (2017) demonstrated that drought stress causes broad metabolic changes in cowpea, with significant accumulation of proline, galactinol, and quercetin derivatives. These compounds acted as osmoprotectants and antioxidants, supporting yield under water‐limited conditions and suggesting their potential as biochemical markers for selecting drought‐tolerant lines. Complementary findings by Gomes et al. (2020) showed that drought‐tolerant landraces accumulate unique metabolites such as sucrose, fucose, alanine, and putrescine, while maintaining stable organic acid profiles, indicating superior metabolic homeostasis. Similarly, Jayawardhane et al. (2022) reported that metabolic adjustments are stress‐specific and organ‐dependent: drought primarily induced flavonoid accumulation in leaves, salinity enhanced antioxidant metabolism in both roots and shoots, while hypoxia suppressed flavonoid levels. Together, these studies underline the dynamic metabolic responses of cowpea and their relevance for breeding resilience to multiple abiotic stresses.

Postharvest metabolomics has also provided important insights into senescence and storage quality. Bai et al. (2024) showed that nitric oxide treatment preserves nutritional quality by enhancing flavonoids, phenolic acids, and alkaloids, while reducing lignin biosynthesis. Similarly, Han et al. (2024) reported that cold storage induces lipid peroxidation and flavonoid accumulation, highlighting both damage‐associated and protective metabolites involved in chilling stress. These findings provide candidate biochemical pathways for improving postharvest management and extending cowpea shelf life.

Metabolomics has also been instrumental in elucidating cowpea defense mechanisms against pests. Early work by Alabi et al. (2006) linked protein and glucose levels in reproductive tissues to resistance against flower bud thrips, while subsequent studies by Alabi et al. (2011) showed that polyphenols and terpenoids in floral structures reduced thrips infestation through direct toxicity. More recent advances have expanded this understanding. Gitonga et al. (2022) highlighted the role of genotype × environment interactions in shaping metabolite accumulation, with resistant lines maintaining stable levels of flavonoids and antioxidants across conditions. Oladejo et al. (2025) further confirmed that proteins, soluble amino acids, and antioxidants serve as key biochemical markers for thrips resistance. Using targeted metabolomics, He et al. (2023, 2025) identified flavonoids such as luteolin and isoflavonoids like coumestrol as direct defense compounds against Megalurothrips usitatus, with clear links to upregulated biosynthetic genes. Similarly, Kpoviessi et al. (2021) demonstrated that phenolic compounds, tannins, and carbohydrates were strongly associated with resistance against the cowpea bruchid, providing biochemical targets for breeding pest‐resistant varieties. These studies emphasize the utility of metabolomics in dissecting natural defense pathways and guiding pest‐resilient cowpea breeding.

Beyond stress and defense, metabolomics has also revealed the nutritional and functional diversity of cowpea. Tsamo et al. (2020) profiled seed coat metabolites of Ghanaian accessions and identified phenolic acids, flavonoids, and anthocyanins, with dark‐colored seeds accumulating the highest levels of bioactive compounds. These findings highlight opportunities for nutritional enhancement and biofortification. Similarly, Karuwal et al. (2023) characterized amino acids, fatty acids, sterols, and phenolics in landraces from Indonesia, uncovering significant biochemical variation among accessions and linking seed pigmentation to antioxidant potential. Such studies demonstrate how metabolomics can uncover hidden nutritional traits and guide the utilization of local genetic resources for food security and health.

## 11 Integrated Omics for Cowpea Improvement

Single‐omics technologies have substantially advanced the understanding of cowpea biology by identifying genes, transcripts, proteins, and metabolites associated with agronomically important traits. However, because each omics technology captures only one level of biological organization, it provides an incomplete representation of the molecular processes underlying complex phenotypes (Hasin et al. 2017; Li et al. 2020). Traits such as drought tolerance, pest and disease resistance, nutritional quality, and yield stability are controlled by intricate interactions among genomic variation, transcriptional regulation, protein activity, metabolite accumulation, and environmental influences rather than from single gens acting independently (Deshmukh et al. 2025; Yan and Wang 2023). Single‐omics analyses frequently identify trait‐associated molecular components but are unable to resolve the regulatory hierarchies and biological interactions that collectively determine phenotype across diverse environments. Integrating genomics, transcriptomics, proteomics, and metabolomics within a systems biology framework overcomes these limitations by reconstructing the molecular continuum from genetic variation to phenotypic expression. This integrative strategy enables the identification of causal genes, regulatory pathways, and molecular biomarkers that underpin complex traits, thereby providing a stronger foundation for precision breeding, genomic selection, and genome editing than any individual omics technology alone (Zhang, Zhang, et al. 2022; Yan and Wang 2023; Yoosefzadeh Najafabadi et al. 2023).

Rather than functioning as independent analytical technologies, genomics, transcriptomics, proteomics, and metabolomics represent complementary layers of biological information that collectively describe the progression from genotype to phenotype. Genetic variation establishes the repertoire of functional alleles, transcriptomic regulation determines when and where genes are expressed, proteomic dynamics define cellular functions through protein abundance and post‐translational modifications, and metabolomic profiles capture the biochemical state ultimately expressed as physiological performance, nutritional quality, and stress adaptation. Integrating these molecular datasets with high‐throughput phenotyping and environmental information enables the reconstruction of gene regulatory networks, identification of expression quantitative trait loci (eQTLs), and discovery of biomarkers and characterization of genotype × environment (G × E) interactions governing complex traits (Li et al. 2020; Yan and Wang 2023). Artificial intelligence (AI) and machine learning (ML) further strengthen this system‐level framework by enabling the integration of high‐dimensional and heterogeneous biological datasets, facilitating feature selection, dimensionality reduction, predictive modeling, and genomic prediction of complex quantitative traits that are difficult to resolve using conventional statistical approaches (Yan and Wang 2023; Yoosefzadeh Najafabadi et al. 2023). Collectively, these technologies establish integrated multi‐omics as a predictive breeding framework capable of accelerating candidate gene discovery, genomic selection, and genome editing for the development of climate‐resilient and nutritionally enhanced cowpea cultivars.

**FIGURE 3:**
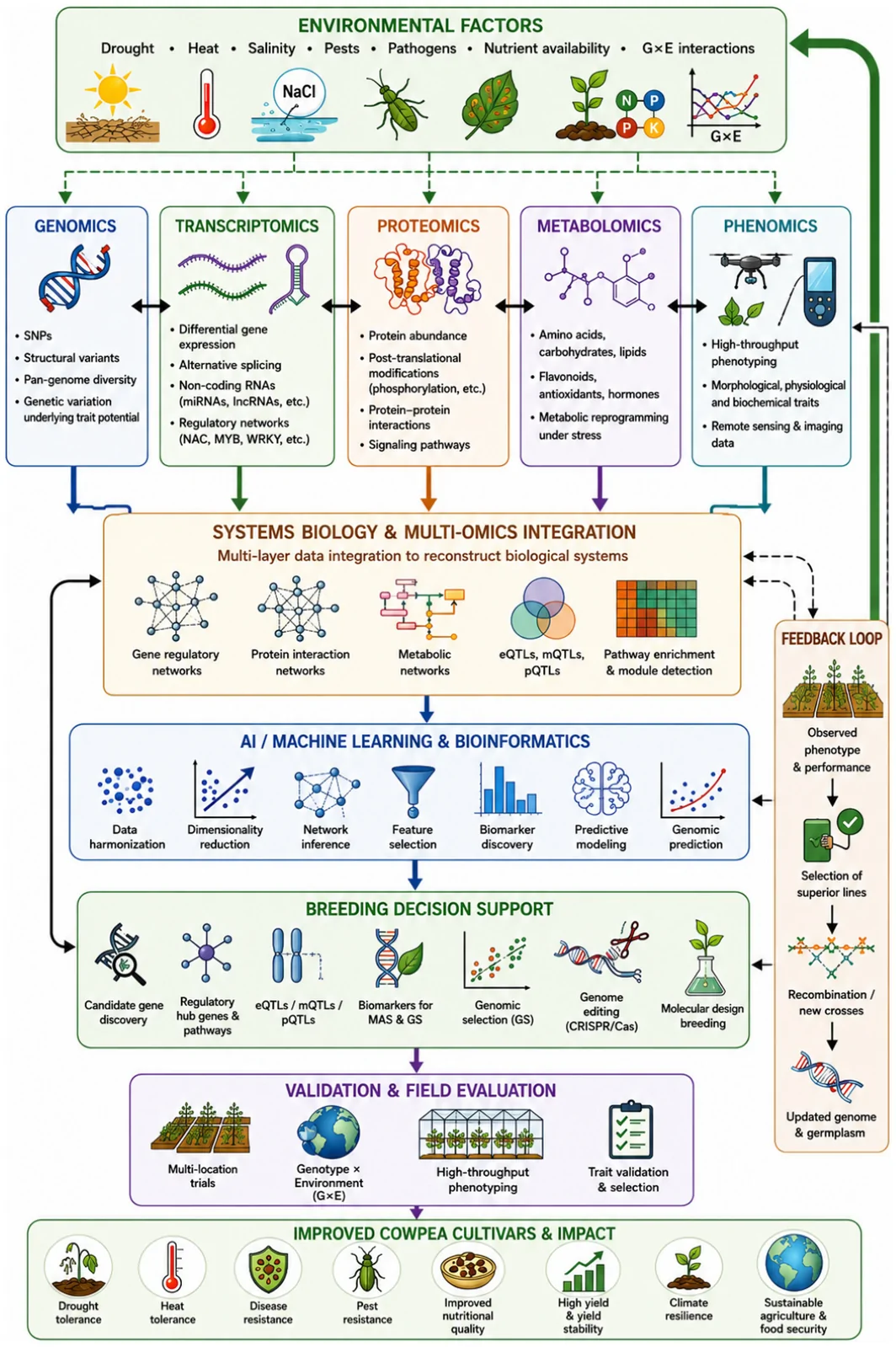
Integrated multi‐omics breeding framework for cowpea improvement: Linking genotype to phenotype through systems biology and artificial intelligence. This framework illustrates the hierarchical progression from environmental stimuli to molecular responses and breeding applications in cowpea. Environmental factors, including drought, heat, salinity, pests, pathogens, nutrient availability, and genotype × environment (G × E) interactions, influence successive molecular layers comprising genomics, transcriptomics, proteomics, metabolomics, and phenomics. These datasets are integrated through systems biology approaches to reconstruct regulatory networks, identify biological pathways, detect quantitative molecular biomarkers (eQTLs, mQTLs, and pQTLs), and model genotype‐to‐phenotype relationships. Artificial intelligence (AI) and machine learning (ML) algorithms facilitate data harmonization, feature selection, dimensionality reduction, network inference, biomarker discovery, genomic prediction, and predictive modeling to support evidence‐based breeding decisions. The resulting outputs guide molecular breeding, genomic selection, genome editing, and trait validation across multiple environments through an iterative breeding cycle that continuously refines selection accuracy. Collectively, this framework provides a systems‐level strategy for accelerating the development of climate‐resilient, disease‐resistant, high‐yielding, and nutritionally improved cowpea cultivars. The framework was created using BioRender based on a systematic literature of Web of Science and Scopus databases.

We propose a conceptual integration framework for cowpea in which multi‐omics data are organized into a hierarchical continuum linking genotype to phenotype under environmental influence. At the base, genomics defines the repertoire of genetic variation (SNPs, structural variants, pan‐genome diversity) that establishes trait potential. Transcriptomics captures condition‐specific regulation of this potential through differential expression, alternative splicing, and non‐coding RNA activity. Proteomics translates regulatory signals into functional units by defining protein abundance, modifications, interactions, and pathway activity. Metabolomics reflects the biochemical output of these processes, determining physiological performance, nutritional quality, and stress adaptation. Phenomics and environmental data provide the contextual layer that links molecular states to observable traits under specific agroecological conditions. In this framework, integration is not merely the concatenation of datasets but the reconstruction of causal chains: genetic variants → regulatory networks → protein functions → metabolic states → phenotypic outcomes, modulated by environment. This conceptual model provides a structured basis for hypothesis‐driven discovery, biomarker development, and predictive breeding. This hierarchical continuum is summarized in Figure 3, which illustrates how environmental stimuli modulate successive omics layers and how integrated datasets, strengthened by systems biology and AI/ML approaches, converge to explain genotype‐to‐phenotype relationships and guide predictive breeding in cowpea.

Building on the conceptual framework illustrated in Figure 3, the practical integration of multi‐omics for cowpea breeding can be structured into four analytical steps: (1) data harmonization, where multi‐omics datasets are standardized, normalized, and linked to common genetic references (e.g., IT97K‐499‐35 or pan‐genome coordinates); (2) network reconstruction, where gene–protein–metabolite relationships are modeled using correlation, causality inference, and machine learning to build gene regulatory and metabolic networks; (3) trait modeling, where these networks are integrated with phenotypic and environmental data to identify expression quantitative trait loci (eQTLs), metabolite‐based QTLs (mQTLs), and key regulatory nodes; and (4) predictive deployment, where the resulting models are used for genomic selection, candidate gene prioritization, and genome editing target definition. This framework transforms multi‐omics from a descriptive tool into a predictive engine for breeding, enabling the selection of genotypes with optimized regulatory and metabolic configurations for resilience, yield, and nutritional quality.

The value of integrated multi‐omics is increasingly evident in cowpea research, where the combined analysis of transcriptomic, proteomic, and metabolomic datasets has provided mechanistic insights into complex traits that could not be resolved through single‐omics approaches. Across studies, integrated omics has consistently revealed coordinated regulatory networks linking gene expression, protein function, and metabolite accumulation during responses to biotic and abiotic stresses, thereby improving the understanding of stress adaptation and trait regulation. For example, in line with the proposed integration framework, transcriptome–metabolome integration demonstrated that melatonin‐mediated delay of postharvest senescence was associated with coordinated modulation of genes involved in amino acid and carbohydrate metabolism, hormone signaling, and antioxidant pathways, resulting in enhanced metabolite accumulation and delayed tissue deterioration (Liu et al. 2024). Similarly, consistent with the regulatory–metabolic axis of the framework, the integration of genomic and transcriptomic analyses revealed that microRNA‐mediated regulation of Argonaute (AGO) proteins, glutathione‐S‐transferase (GST), and other defense‐related genes plays a central role in antiviral responses against Cowpea severe mosaic virus (CPSMV), highlighting the importance of post‐transcriptional regulatory networks in disease resistance (Martins et al. 2020).

Complementary proteomic and metabolomic analyses further demonstrated that resistance to *Macrophomina phaseolina* is accompanied by extensive metabolic reprogramming, including enhanced antioxidant metabolism, improved photosynthetic performance, and nutritional enrichment, thereby illustrating the close relationship between plant defense and metabolic regulation (Ahmad et al. 2020). These findings illustrate how regulatory networks at the transcript level are translated into functional protein and metabolic states, a theme that is further elaborated in proteomic–metabolomic studies of fungal resistance. Likewise, integrated transcriptomic and metabolomic profiling showed that pesticide exposure induces coordinated transcriptional and metabolic perturbations affecting carbohydrate, amino acid, lipid, and flavonoid metabolism, providing molecular evidence of how environmental stress reshapes cellular homeostasis in cowpea (Zhang, Yin, et al. 2022).

Across these studies, integrated omics consistently reveals a recurring architecture of stress and quality responses in cowpea: (i) early transcriptional reprogramming of hormone signaling (ABA, JA) and stress‐responsive transcription factors (NAC, MYB, WRKY); (ii) downstream modulation of protein activity and post‐translational regulation (e.g., AGO proteins, GST, antioxidant enzymes); and (iii) coordinated metabolic adjustments in amino acids, carbohydrates, flavonoids, and antioxidants that determine physiological performance and nutritional quality. Whether in postharvest senescence, antiviral defense, fungal resistance, or pesticide stress, the same regulatory modules appear to be rewired in a coordinated manner, suggesting that multi‐omics can uncover core, reusable stress‐response networks rather than isolated, trait‐specific signals. This pattern supports the proposed integration framework, where genotype‐dependent regulatory networks are translated into protein and metabolic states that jointly determine phenotype under environmental constraints. By mapping these recurrent modules across traits and stresses, integrated omics shifts the focus from single‐marker associations to system‐level drivers of resilience and quality. Collectively, these studies demonstrate that integrated multi‐omics not only identifies candidate genes associated with economically important traits but also explains the regulatory networks and biochemical pathways that underpin stress resilience, nutritional quality, and physiological performance, thereby providing a mechanistic foundation for precision breeding.

The expanding availability of multi‐omics datasets has shifted cowpea research toward data‐intensive systems biology, where extracting biologically meaningful information from heterogeneous datasets has become a major analytical challenge. Integrating genomic, transcriptomic, proteomic, metabolomic, phenomic, and environmental data generates high‐dimensional biological datasets characterized by substantial complexity, making conventional statistical approaches inadequate for capturing the nonlinear interactions underlying complex quantitative traits (Yan and Wang 2023; Yoosefzadeh Najafabadi et al. 2023). Advances in artificial intelligence (AI) and machine learning (ML) have emerged as powerful computational tools for overcoming these limitations by integrating heterogeneous datasets, reducing dimensionality, identifying informative molecular features, and reconstructing gene regulatory networks that link genotype to phenotype (Yan and Wang 2023; Yoosefzadeh Najafabadi et al. 2023). These computational approaches improve candidate gene prioritization, facilitate the identification of expression quantitative trait loci (eQTLs) and metabolite‐associated biomarkers, and enhance genomic prediction for complex traits such as drought tolerance, disease resistance, yield stability, and nutritional quality (Zhang, Zhang, et al. 2022; Yan and Wang 2023). Furthermore, integrating multi‐omics data with high‐throughput phenotyping and genotype × environment (G × E) information enables predictive breeding frameworks capable of accelerating genomic selection, improving selection accuracy across diverse production environments, and supporting the development of climate‐resilient cowpea cultivars (Yoosefzadeh Najafabadi et al. 2023).

## 12 Conclusion and Perspectives

Cowpea ( *Vigna unguiculata* ), a protein‐rich and drought‐tolerant legume widely cultivated across sub‐Saharan Africa and other semi‐arid regions, remains central to food security, nutrition, and smallholder livelihoods. However, its full genetic potential remains only partially exploited because many priority breeding traits, including drought tolerance, pest resistance, and nutritional quality, are quantitatively inherited and strongly influenced by genotype × environment interactions, limiting the efficiency of conventional phenotypic selection for improving these complex traits.

Although omics technologies have substantially advanced the understanding of cowpea biology, their integration into routine breeding programs remains limited. Most studies continue to rely on single omics technology, with relatively few adopting multi‐omics technologies that capture interactions among genes, transcripts, proteins, and metabolites. This gap constrains the translation of molecular discoveries into predictive breeding models and validated molecular markers for large‐scale cultivar development. Realizing the full potential of multi‐omics‐assisted breeding will require stronger integration of existing genomic resources, including the reference genome, pan‐genome, SNP arrays, GWAS panels, and MAGIC populations, with transcriptomic, proteomic, metabolomic, and high‐throughput phenotyping datasets.

Coupling these resources with advanced bioinformatics and artificial intelligence (AI)‐ and machine learning (ML)‐driven predictive breeding frameworks will improve genotype–phenotype prediction and accelerate data‐driven breeding decisions. Integrating genomic selection, genome editing, and metabolite‐assisted selection into multi‐omics‐informed breeding pipelines has the potential to shorten breeding cycles, improve selection accuracy, and unlock the full genetic potential of cowpea. Collectively, these technologies accelerate the development of climate‐resilient, high‐yielding, and nutritionally improved cowpea cultivars, thereby supporting sustainable agricultural production, strengthening food and nutrition security, and improving smallholder resilience under the effects of climate change and rapid population growth.

## Funding

The authors have nothing to report.

## Conflicts of Interest

The authors declare no conflicts of interest.

## Data Availability

The authors have nothing to report.
